# Antiphospholipid antibodies during acute COVID-19 are not associated with long COVID: findings from a retrospective cohort study

**DOI:** 10.3389/fimmu.2025.1634663

**Published:** 2025-08-06

**Authors:** Robin Arcani, Alexandre Brodovitch, Xavier Heim, Jean-Louis Mège, Nathalie Bardin

**Affiliations:** ^1^ Internal Medicine and Therapeutics department, Centre Hospitalo-Universitaire (CHU) La Timone, Assistance Publique-Hôpitaux de Marseille (AP-HM), Marseille, France; ^2^ Center for Cardiovascular and Nutrition research (C2VN), Institut national de recherche pour l'agriculture et l'alimentation (INRA) 1260, Institut national de la santé et de la recherche médicale (INSERM) UMR_S 1263, Aix-Marseille University, Marseille, France; ^3^ Service d’Immunologie, Pôle de Biologie, Biogénopole, Hôpital de la Timone, Assistance Publique-Hôpitaux de Marseille (AP-HM), Marseille, France; ^4^ Aix-Marseille Université, Institut de Recherche pour le Développement (IRD), Microbes, Evolution, Phylogénie et Infection (MEPHI), Institut Hospitalo-Universitaire (IHU)-Méditerranée Infection, Marseille, France

**Keywords:** antiphospholipid antibodies, pathophysiology, autoimmunity, long covid, thrombosis

## Abstract

**Introduction:**

Long COVID is a public health issue with complex pathophysiology, potentially involving immunoinflammatory and prothrombotic mechanisms. Antiphospholipid antibodies (aPL) have been observed in acute COVID-19 and speculated to contribute to long COVID development. Our goal was to determine if the presence of aPL was associated with the progression towards long COVID.

**Methods:**

We retrospectively analyzed all adult patients screened for aPL during acute COVID-19 in our institution between April 2020 and April 2022. Only patients with at least one follow-up ≥3 months post-infection were included.

**Results:**

Among 114 patients (median age 64.0 years, 44.7% female), 19 (16.7%) developed long COVID. Those with long COVID were younger and more frequently admitted to ICU than those who recovered. However, aPL positivity did not differ significantly between patients with and without long COVID (63.2% vs. 66.3%, p = 0.79).

**Conclusion:**

Our findings suggest no association between aPL and the development of long COVID. Prior associations may reflect confounding factors such as ICU admission.

## 1 Introduction

Long COVID, also called “chronic COVID-19” or “post-COVID-19 condition,” represents a major global health challenge, with an estimated cumulative global incidence of 400 million individuals and an annual economic burden approaching $1 trillion (1). The persistent sequelae and long-term complications of COVID-19 may result from various putative pathophysiological mechanisms. Among these, immunoinflammatory mechanisms and immunothrombosis have been proposed as key contributors to the pathogenesis of long COVID (2).

Antiphospholipid autoantibodies (aPL)—including lupus anticoagulant (LA), IgG and/or IgM anti-cardiolipin autoantibodies (aCL), and IgG and/or IgM anti-β2 glycoprotein I antibodies (aβ2GPI)—are central to the diagnosis of antiphospholipid syndrome (APS), an autoimmune thrombotic disorder (3). These autoantibodies are also considered as vascular risk factors via immunoinflammatory mechanisms (4, 5). Previous studies have reported an association between the presence of aPL during acute COVID-19 and disease severity (6, 7).

Furthermore, our team and others (8, 9) have suggested a potential link between aPL and the development of long COVID. We previously reported the case of a 58-year-old woman with long COVID, characterized by persistent aCL positivity over one year and presenting with unexplained fatigue, insomnia, headache, and chronic memory impairment since the onset of COVID-19. This case illustrates the possibility that sustained immune activation may be one of the mechanisms underlying long COVID.

To further investigate this hypothesis, we conducted a retrospective cohort study to assess whether the presence of aPL during acute COVID-19 is associated with the development of long COVID.

## 2 Method

### 2.1 Patients

**Figure 1:**
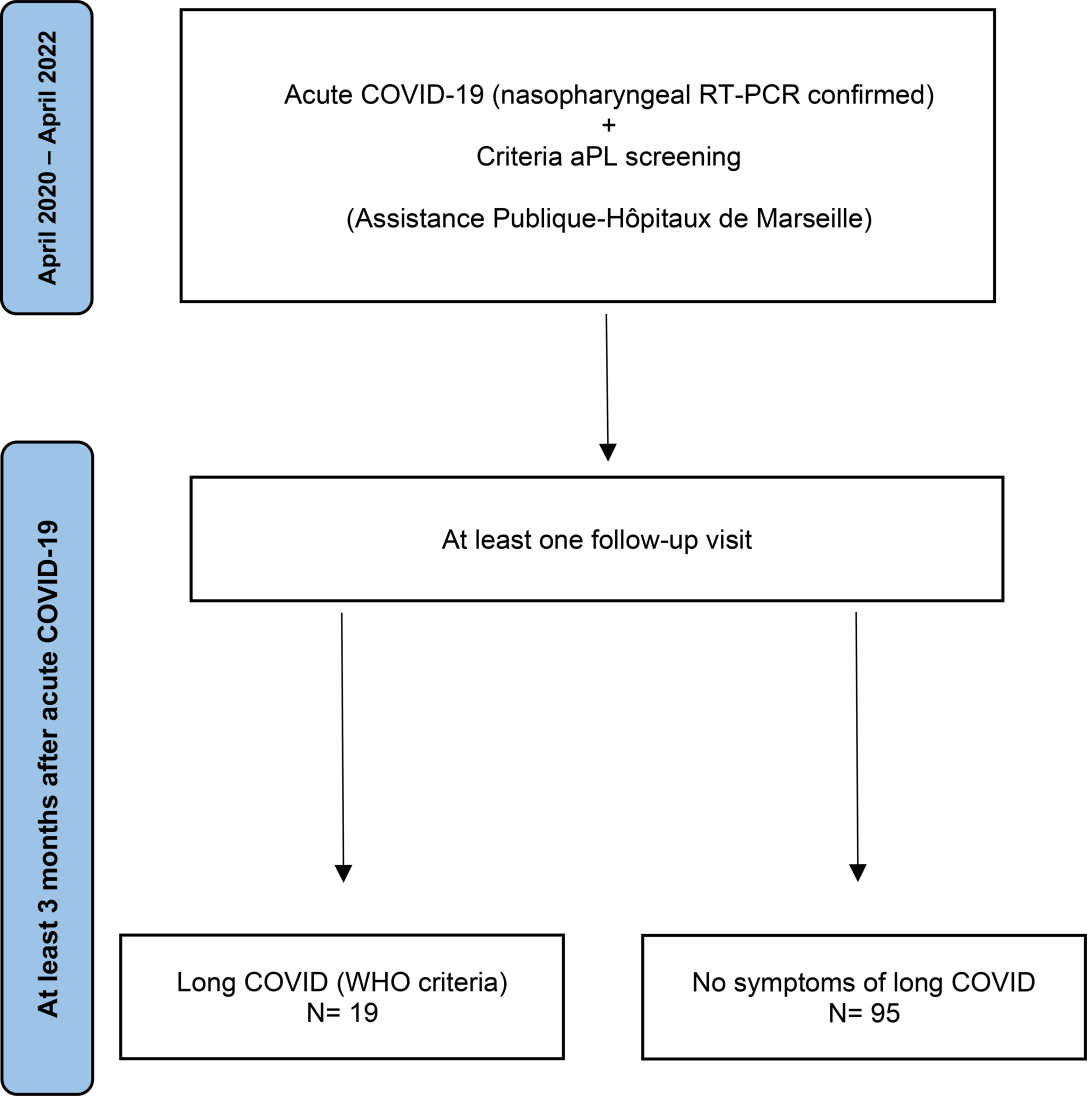
Flow diagram of patients’ selection.

We conducted a retrospective study on all adult patients who underwent criteria aPL screening (LA, IgG and IgM aCL, IgG and IgM aβ2GPI) during the acute phase of COVID-19 (confirmed by nasopharyngeal RT-PCR) between April 2020 and April 2022 at our institution (Assistance Publique–Hôpitaux de Marseille, France) and who had at least one follow-up visit more than three months after the acute infection (**Figure 1**). Patients were classified as having long COVID if they had a history of SARS-CoV-2 infection confirmed by RT-PCR within the previous three months before symptom onset, with symptoms lasting at least two months and not explained by an alternative diagnosis, according to the WHO definition (10). Clinical, biological, and follow-up data of these patients were collected from electronic medical records.

### 2.2 Biological data

aPL concentrations were measured using commercially available ELISA kits. IgG/IgM aCL and aβ2GPI were measured on sera using Cardiolisa^®^ (Theradiag, Marne-la-Vallée, France) and Orgentec Diagnostika^®^ (Mainz, Germany), respectively. According to manufacturer recommendations and in-house validation, positivity cut-offs were 15 U/mL for aCL and 8 U/mL for aβ2GPI. To minimize nonspecific binding, all positive samples were tested in duplicate, and background absorbance from uncoated wells was subtracted from the coated well optical density. We use the cut-off provided by the kit supplier. These cut-offs were confirmed by using a control group (composed by healthy controls from blood donation).

LA was assessed on blood following ISTH guidelines using two clotting assays: the Partial Thromboplastin Time-Lupus Anticoagulant (PTT-LA, Diagnostica Stago^®^, Asnières-sur-Seine, France) and the Dilute Russell Viper Venom Time (dRVVT, Hyphen BioMed^®^, Neuville-sur-Oise, France). A positive lupus anticoagulant was defined as simultaneous positivity of both assays. The Rosner Index (RI) was considered positive when >15. The dRVVT was interpreted as positive when the normalized ratio (NR) exceeded 1.2. The anti-Xa activity was systematically checked on all samples. When anti-Xa activity was positive, the LA result was reported as uninterpretable.

The aPL screening was performed reproducibly for all patients, using the same assays ensuring comparability across the cohort.

### 2.3 Ethics

The study was approved by the Institutional Review Board of Assistance Publique–Hôpitaux de Marseille (GDPR approvals: PADS21–4 and PADS22-15) and conducted in accordance with the Declaration of Helsinki.

### 2.4 Statistical analysis

Quantitative variables were described using medians and interquartile ranges (IQR), while categorical variables were described using numbers and percentages. Quantitative data were compared using the Student’s t- or Mann-Whitney U test, while qualitative data were compared with the Chi-square or Fisher’s exact test when appropriate. The tests were two-sided. P-values <0.05 were considered significant. All analyses were performed with R software (R Foundation for Statistical Computing, Vienna, Austria).

## 3 Results

During the acute COVID-19, detection of aPL (LA, IgG and IgM aCL, IgG and IgM aβ2GPI) was performed in 114 patients who had at least one follow-up visit more than 3 months after the acute COVID-19 (**Table 1**). Among them, 51 (44.7%) were female, and the median age was 64.0 years (IQR: 55-75). Because of the severity of COVID-19, forty-seven patients (41.2%) were hospitalized during the acute phase of COVID-19 and 31 patients (27.2%) were admitted to the intensive care unit (ICU).

**Table 1:** Comparison between cured COVID and long COVID.

	Covid (N=95)	Long Covid (N=19)	Total (N=114)	P value
Sex – F (%)	41 (43.2)	10 (52.6)	51 (44.7)	0.45^1^
Age - Median (IQR)	67.0 (56-79.5)	58.0 (49.5-64.5)	64.0 (55-75)	0.020^2^
Antiphospholipid positivity – N (%) Lupus Anticoagulant – N (%) Anticardiolipin IgG – N (%) Anticardiolipin IgM – N (%) Anti-β2GPI IgG – N (%) Anti-β2GPI IgM – N (%)	63 (66.3) 26 (27.4) 29 (30.5) 9 (9.5) 7 (7.4) 13 (13.7)	12 (63.2) 2 (10.5) 6 (31.6) 4 (21.1) 1 (5.3) 2 (10.5)	75 (65.8) 28 (24.6) 35 (30.7) 13 (11.4) 8 (7.0) 15 (13.2)	0.79^1^ 0.12^1^ 0.93^1^ 0.15^1^ 0.74^1^ 0.71^1^
Antinuclear antibodies positivity – N (%)	9/20 (45.0)	5/11 (45.5)	14/31 (45.2)	0.98^1^
Admission to the intensive care unit – N (%)	21 (22.1)	10 (52.6)	31 (27.2)	0.006^1^
Hospitalization for SARS-CoV-2 infection at the acute COVID-19	34 (35.8)	13 (68.4)	47 (41.2)	0.008^1^
Comorbidities Previous history of thrombosis – N (%) Previous history of stroke – N (%) Diabetes – N (%) High blood pressure – N (%) Coronary heart disease – N (%) Chronic kidney disease – N (%) Chronic respiratory disease – N (%) Cancer – N (%)	10/92 (10.9) 6/92 (6.5) 23/92 (25.0) 47/92 (51.1) 11/92 (12.0) 12/92 (13.0) 11/93 (11.8) 7/92 (7.6)	1/18 (5.6) 1/18 (5.6) 2/18 (11.1) 4/18 (22.2) 0/18 (0) 0/18 (0.0) 2/18 (11.1) 0/18 (0)	11/110 (10.0) 7/110 (6.4) 25/110 (22.7) 51/110 (46.4) 11/110 (10.0) 12/110 (10.9) 13/111 (11.7) 7/110 (6.4)	0.49^1^ 0.88^1^ 0.20^1^ 0.025^1^ 0.12^1^ 0.11^1^ 0.93^1^ 0.23^1^
Hydroxychloroquine treatment – N (%)	35/92 (38.0)	13/17 (76.5)	48/109 (44.0)	0.003^1^

1. Pearson’s Chi-squared test.

2. Student t-test.

Seventy-five patients (65.8%) were tested positive for at least one aPL during acute phase of COVID-19. We found LA, aCL IgG, aCL IgM, aβ2GPI IgG and aβ2GPI IgM in 24.6%, 30.7%, 11.4%, 7.0% and 13.2% of the patients, respectively.

Nineteen patients (16.7%) developed long COVID during follow-up. Compared to patients who recovered, those who developed long COVID were more likely to have been hospitalized (68.4% vs. 35.8%, p= 0.008), to have required ICU care (52.6% vs. 21.1%, p= 0.006) and were significantly younger (median age: 58.0 vs. 67.0 years, p= 0.020). They were also more frequently treated with hydroxychloroquine during the acute phase of COVID-19 (76.5% vs. 38.0%, p = 0.003). In 48 patients who received hydroxychloroquine, 29 were hospitalized for acute COVID-19 (60.4%) whom 17 were hospitalized in ICU (35.4%). Patients who did not receive hydroxychloroquine were less hospitalized (29.5%) and have less required ICU care (23.0%).

However, the prevalence of aPL positivity did not differ significantly between patients who developed long COVID and those who did not (63.2% vs. 66.3%, p = 0.79). No statistically significant differences were observed for any individual aPL subtype between the two groups.

We assessed the prevalence of aPL, at least 12 weeks after the initial screening, in 55 patients (23 who developed long COVID and 32 who did not). aPL positivity was found in 7 of 23 patients with long COVID (30.4%) compared to 12 of 32 patients without long COVID (37.5%). This difference was not statistically significant (p = 0.59).

Among the patients who were screened positive during the acute phase of COVID-19, 45 underwent repeat aPL screening at least 12 weeks after the initial measurement. Among them, aPL positivity was found in 5 of 13 patients with long COVID (38.5%) compared to 12 of 32 patients without long COVID (37.5%). This difference was not statistically significant (p = 0.95).

## 4 Discussion

We present the largest cohort of patients screened for aPL during acute COVID-19 and followed more than three months later to assess their progression to long COVID. Our findings do not support a role for aPL in the pathogenesis of long COVID. The links between aPL and long COVID found in previous studies (9) are potentially a statistical relationship related to the higher presence of aPL in patients who were admitted to the ICU (6) and to the association between long COVID and ICU admission, as we have just demonstrated here. It therefore does not appear that aPL screening should be considered as a useful marker for diagnosing or predicting the occurrence of long COVID. This is not surprising, as these antibodies, although present in both acute COVID-19 and long COVID, are not consistently associated with clinical events such as thrombosis (7, 11).

The incidence of long COVID in our cohort is consistent with that reported in other studies (ranging from 10% to 35%, and up to 56.9% in some series) (12, 13), although more recent data seem to suggest a lower incidence, in the range of just a few percent (2). This wide variability is mainly explained by the diagnostic criteria used to define long COVID, highlighting the need for robust and internationally recognized definitions.

In our cohort, we observed a higher incidence of long COVID compared to other recent studies, likely because a significant proportion of our patients presented with severe forms of the disease requiring admission to the ICU, as our study was conducted in a tertiary university hospital that primarily admitted the most critically ill patients.

Moreover, the incidence of aPL positivity in our cohort is relatively high but remains within the range of incidences reported in the literature (from 35 to 92% in ICU) (6, 14, 15). In our cohort, it is probably due to the high prevalence of patients with severe COVID-19.

In addition, we found a statistical link between hydroxychloroquine use and progression to long COVID. However, this cannot be definitively established due to the retrospective design of our study and the little number of patients treated with hydroxychloroquine. Moreover, patients who received hydroxychloroquine were more severe than those who did not. The severity of acute COVID-19 is a well-known risk factor to develop long COVID. Furthermore, we did not perform a multivariate analysis to neutralize potential confounding bias, this finding should be interpreted with caution. Nevertheless, these data support the need for further exploration of the potential impact of acute-phase treatments on the risk of long COVID. For instance, antiviral therapies have been associated with a reduced incidence of long COVID (9). This is an important issue, as it could influence therapeutic decisions during the acute COVID-19, which continues to affect many people worldwide.

Our study has some limitations. Its retrospective design and limited sample size constrain the power and the generalizability of our findings. These results should therefore be considered exploratory. Nonetheless, it remains the largest cohort exploring the association between aPL and long COVID. We would have wished to investigate the association between aPL positivity and the different types of symptoms experienced by patients with long COVID. Unfortunately, due to the small number of patients with long COVID in our cohort, the analyses lacked statistical significance. These analyses would have been particularly interesting, as the symptoms reported by patients with long COVID are highly heterogeneous, suggesting that the underlying pathophysiological mechanisms may differ depending on the organ systems involved. Larger studies will be necessary to further explore this question.

Finally, the lack of a control group precludes definitive exclusion of the involvement of aPL and immunoreactivity in other post-acute infectious syndromes, warranting further comprehensive investigations.

## 5 Conclusion

Our study adds to the growing body of evidence suggesting that aPL are not implicated in the pathogenesis of long COVID. These findings argue against the routine use of aPL screening to predict long COVID risk.

## Data Availability

The raw data supporting the conclusions of this article will be made available by the authors, without undue reservation.

## References

[1] Al-Aly Z Davis H McCorkell L Soares L Wulf-Hanson S Iwasaki A. Long COVID science, research and policy. Nat Med. (2024) 30:2148–64. doi: 10.1038/s41591-024-03173-6, PMID: 39122965

[2] Greenhalgh T Sivan M Perlowski A Nikolich JŽ. Long COVID: a clinical update. Lancet. (2024) 404:707–24. doi: 10.1016/S0140-6736(24)01136-X, PMID: 39096925

[3] Barbhaiya M Zuily S Naden R Hendry A Manneville F Amigo M-C. 2023 ACR/EULAR antiphospholipid syndrome classification criteria. Ann Rheum Dis. (2023) 82:1258–70. doi: 10.1136/ard-2023-224609, PMID: 37640450

[4] Musiał J. Antiphospholipid antibodies and thrombosis. Thromb Res. (2012) 129:345–7. doi: 10.1016/j.thromres.2011.10.029, PMID: 22119156

[5] Lambert M Brodovitch A Mège J-L Bertin D Bardin N. Biological markers of high risk of thrombotic recurrence in patients with antiphospholipid syndrome: A literature review. Autoimmun Rev. (2024) 23:103585. doi: 10.1016/j.autrev.2024.103585, PMID: 39094811

[6] Bertin D Brodovitch A Lopez A Arcani R Thomas GM Beziane A. Anti-cardiolipin IgG autoantibodies associate with circulating extracellular DNA in severe COVID-19. Sci Rep. (2022) 12:12523. doi: 10.1038/s41598-022-15969-y, PMID: 35869087 PMC9305055

[7] Arcani R Cauchois R Suchon P Weber S Jean R Jarrot P-A. True” Antiphospholipid syndrome in COVID-19: contribution of the follow-up of antiphospholipid autoantibodies. Semin Thromb Hemost. (2023) 49:97–102. doi: 10.1055/s-0042-1758118, PMID: 36335917

[8] Bertin D Kaphan E Weber S Babacci B Arcani R Faucher B. Persistent IgG anticardiolipin autoantibodies are associated with post-COVID syndrome. Int J Infect Dis. (2021) 113:23–5. doi: 10.1016/j.ijid.2021.09.079, PMID: 34614444 PMC8487460

[9] Pisareva E Badiou S Mihalovičová L Mirandola A Pastor B Kudriavtsev A. Persistence of neutrophil extracellular traps and anticardiolipin auto-antibodies in post-acute phase COVID-19 patients. J Med Virol. (2023) 95:e28209. doi: 10.1002/jmv.28209, PMID: 36226380 PMC9874393

[10] Soriano JB Murthy S Marshall JC Relan P Diaz JV WHO Clinical Case Definition Working Group on Post-COVID-19 Condition. A clinical case definition of post-COVID-19 condition by a Delphi consensus. Lancet Infect Dis. (2022) 22:e102–7. doi: 10.1016/S1473-3099(21)00703-9, PMID: 34951953 PMC8691845

[11] Emmenegger M Emmenegger V Shambat SM Scheier TC Gomez-Mejia A Chang C-C. Antiphospholipid antibodies are enriched post-acute COVID-19 but do not modulate the thrombotic risk. Clin Immunol. (2023) 257:109845. doi: 10.1016/j.clim.2023.109845, PMID: 37995947

[12] Di Gennaro F Belati A Tulone O Diella L Fiore Bavaro D Bonica R. Incidence of long COVID-19 in people with previous SARS-Cov2 infection: a systematic review and meta-analysis of 120,970 patients. Intern Emerg Med. (2023) 18:1573–81. doi: 10.1007/s11739-022-03164-w, PMID: 36449260 PMC9709360

[13] Huerne K Filion KB Grad R Ernst P Gershon AS Eisenberg MJ. Epidemiological and clinical perspectives of long COVID syndrome. Am J Med Open. (2023) 9:100033. doi: 10.1016/j.ajmo.2023.100033, PMID: 36685609 PMC9846887

[14] Foret T Dufrost V Salomon Du Mont L Costa P Lefevre B Lacolley P. Systematic review of antiphospholipid antibodies in COVID-19 patients: culprits or bystanders? Curr Rheumatol Rep. (2021) 23:65. doi: 10.1007/s11926-021-01029-3, PMID: 34218350 PMC8254447

[15] Taha M Samavati L. Antiphospholipid antibodies in COVID-19: a meta-analysis and systematic review. RMD Open. (2021) 7:e001580. doi: 10.1136/rmdopen-2021-001580, PMID: 33958439 PMC8103564

